# A Cooperative Shared Control Scheme Based on Intention Recognition for Flexible Assembly Manufacturing

**DOI:** 10.3389/fnbot.2022.850211

**Published:** 2022-03-16

**Authors:** Guangbing Zhou, Jing Luo, Shugong Xu, Shunqing Zhang

**Affiliations:** ^1^School of Information and Communication Engineering, Shanghai University, Shanghai, China; ^2^Institute of Intelligent Manufacturing, Guangdong Academy of Sciences, Guangzhou, China; ^3^South China Robotics Innovation Research Institute, Foshan, China; ^4^School of Automation, Wuhan University of Technology, Wuhan, China

**Keywords:** cooperative shared control, intention recognition, obstacle avoidance, human-robot collaboration, assembly task

## Abstract

Human–robot collaboration (HRC) has been widely utilized in industrial manufacturing and requires a human to cooperate with a robot at the same workspace. However, as HRC focuses on workspace sharing along with independent work, it is not a real collaboration between a human and a robot and, thus, cannot guarantee a smooth cooperation and synchronous operation. To this end, a cooperative shared control scheme based on intention recognition is proposed in this study by sharing workspace and time. In the proposed method, a classification algorithm based on three-dimensional (3D) point cloud is utilized to recognize the human operation intention. Then, the robot can select a suitable tool to match the human's operation. A robot motion control algorithm is developed to detect the obstacles in the HRC process. A cooperative control strategy is introduced to achieve synchronous operation. A simple assembly task is also performed to demonstrate the proposed scheme's effectiveness. The proposed HRC method with shared control can be extended to more complicated and delicate flexible tasks in assembly manufacturing.

## Introduction

With the rapid development of robotics, robots are being widely utilized in industrial manufacturing. In traditional manufacturing enterprises, a robot plays a significant role in large-scale and smooth production (Su et al., 2020; Luo et al., 2021). This promotes a robot's application to replace a human's monotonous, repetitive work. In this sense, a robot brings advantages such as a higher production rate, lower production cost, and improved economic efficiency into traditional production. However, given the flexible processes of intelligent manufacturing and the complexity of a robot's operating environment, traditional production modes cannot meet the requirements of flexible manufacturing (Luo et al., 2019a; Su et al., 2021). Thus, a production mode with human–robot interaction (PM-HRI) is inevitable. It should be noted that PM-HRI does not replace humans; rather, it is a new type of production based on human–robot collaboration (HRC). PM-HRI can be divided into two types: sharing of workspace and sharing of workspace and time. Nowadays, sharing of workspace is primarily being used. It focuses on the sharing of workspace along with independent work. Compared with the traditional industrial production, robots and the humans working in a shared space can ensure human safety through collaboration, improve production efficiency, and minimize errors, so that humans can focus on more valuable work to achieve complementary advantages. PM-HRI has been successfully applied to human–robot handling, human–robot assembly, and other flexible operations. As mentioned before, PM-HRI does not imply a real cooperation. It cannot guarantee a smooth cooperation and synchronous operation between a human and a robot in a shared workspace. Therefore, it is essential to achieve a shared control between a human and a robot through cooperation. To understand PM-HRI, we will introduce the existing scenarios in terms of shared control (Boessenkool et al., 2011; Jiang et al., 2016), intention recognition (Khoramshahi and Billard, 2019; Jin and Pagilla, 2020), and cooperative control (Pellegrinelli et al., 2016; Yu et al., 2019).

Compared with fully automatic assembly line, the robots are cooperated with the human in a sharing workspace for HRC tasks. It is essential to consider safety and how to allocate the control stratagem between the robot and the human. For a HRC task, it is difficult to enhance the efficiency of the task execution and interaction. In this sense, knowing how to allocate roles and human controls is essential to maximizing the advantages of human involvement in flexible manufacturing (Luo et al., 2019b). Shared control is a solution that enables the human and the robot to work in a sharing workplace and to allocate the control authority according to the interaction profile such that to share the responsibilities of task. Yu et al. (2015) proposed a shared control method to achieve the allocation of robot autonomy and human assistance by fusing human intelligence. Boessenkool et al. (2012) designed a human-in-the-loop control method to improve transparency in terms of a human's task completion time, control effort, and operator cognitive workload. Based on a demonstration, Pérez-del-Pulgar et al. (2016) introduced a forced control method to provide guidance and feedback during a peg-in-hole insertion task. Considering the improved complexity in human management, Ramacciotti et al. (2016) presented a shared control method to compensate and couple human intelligence for industrial robots. In human–robot cooperation, the robots cannot ignore the influence of an obstacle and communication delay. To address this issue, Storms et al. (2017) presented a new predictive model based on shared control for teleoperating mobile robots. Van Oosterhout et al. (2013) developed a haptic shared control algorithm to improve the performance of hot-cell remote handling with controller inaccuracies. To evaluate the user performance in industrial manufacturing, Abi-Farraj et al. (2018) presented a shared control architecture to provide a haptic feedback for the feasibility of user control for evaluation. Additionally, Amirshirzad et al. (2019) proposed a human adaptation approach to instantiate the shared control method in a ball balancing HRC task. O'Keeffe et al. (2016) developed a high level of shared control to allocate authority to the robot and the human and improve the task performance in a multirobot system. Further, Fang et al. (2018) and Islam et al. (2018) presented optimization- and impedance-based shared control methods to facilitate the interaction between robots in a multirobot system. In addition, cooperative control methods, such as learning-based hierarchical control (Deng et al., 2018), forced control (Al-Yacoub et al., 2021), and neuroadaptive cooperative control (Zhang et al., 2018), have been utilized in HRC manipulation. Some research achievements showed that the allocation of control authority can be summarized as a game issue and it can use game-theoretical theory to be addressed. Musić and Hirche (2020) proposed a differential game-theoretic approach with shared control to perform HRC haptic task based on Nash equilibrium optimal solution. In order to provide a systematic methodology to achieve the versatile behaviors between the contact robots and the humans, Li et al. (2016, 2019) developed an interactive controller based on differential game theory and observer. In addition, it is a key to recognize the human's intentions in order to achieve effective HRC. It has been demonstrated that a robot can recognize the human operation intention for synchronous working (Jain and Argall, 2018; Yang et al., 2018). With the development of artificial intelligence, neural networks have been utilized to recognize the human intention in HRC, which include long short-term memory (Yan et al., 2019), radial basis function neural networks (Liu et al., 2019), and recurrent neural networks (Zhang et al., 2020). Jain and Argall (2018) presented a recursive Bayesian filtering algorithm to model the human agents behavior with multiple nonverbal observations. Tanwani and Calinon (2017) learned a generative model to capture the human intention and provide assistance through shared control or autonomous control algorithms. Owing to their favorable performance, researchers have proposed the hidden Markov model (HMM)-based algorithms to estimate the human intention for assembly task (Berg et al., 2018), pick-and-place task (Fuchs and Belardinelli, 2021), and a safe and flexible robotized warehouse (Petković et al., 2019). Guaranteeing the efficiency and safety of HRC by accurately estimating the human control intention is critical for PM-HRI. Liu et al. (2019) developed a deep learning method to predict the human motion intention with context awareness of the manufacturing process. Generally, electromyography (EMG) signals can be utilized to profile the interactions between a human and a robot, which, in a sense, reflect the human operation intention (Peternel et al., 2018). For example, Sirintuna et al. (2020) utilized EMG signals to detect human motion by collaborating with a KUKA LBR cobot. Detecting the human intention in cooperative telemanipulation is challenging and an augmentation algorithm of haptic intention was presented by Panzirsch et al. (2017) to help the human operators in a cooperation task. Further, new collaborative system designs based on HMM and augmented reality have been developed to predict the assembly intention of humans in manufacturing. Additionally, the learning-from-demonstration-based control methods are utilized to achieve seamlessly interaction in the unstructured environments by considering new situations with different positions of objects or poses of users (Rozo et al., 2016). What is more, some researchers learn shared control through the HRC task level. For example, Jin and Pagilla (2021) developed a method based on subgoal identification and adjustment to finish the shared control task in the dynamically changing environments. The abovementioned algorithms are feasible and can successfully recognize a human intention. However, they can only achieve cooperative work through a sharing of workspace and not through a sharing of workspace and time. Furthermore, it is a challenge to achieve active interaction and collaboration in the sharing space and time.

Considering the abovementioned analyses, cooperative work through the sharing of workspace and time between humans and robots is difficult. To this end, this study develops a new HRC scheme for human motion recognition and shared control. In the developed algorithm, the robot can recognize the human operation intention through a classifier and employ a shared control based on an obstacle avoidance strategy to improve the cooperative control performance. The experimental results of an assembly task verify our proposed scheme's effectiveness. The proposed method presents a single collaboration framework to achieve cooperative work under sharing the workspace and time by combining shared control and intention recognition.

The remainder of this article is organized as follows. Section Methods presents the proposed algorithms in terms of the robot system description, integrated framework, intention recognition, and cooperative shared control. Section Results describes the experimental results of the assembly task. The discussion and conclusion are given in Sections Discussion and Conclusion, respectively.

## Methods

### Description of the Robot System

**Figure 1:**
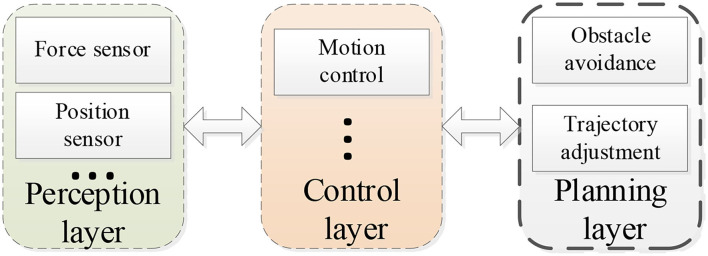
Hierarchical structure of the robot system.

To achieve a safe and effective interaction between a robot and a human in flexible assembly manufacturing, a hierarchical structure is developed for the robot. Figure 1 shows the hierarchical structure. There are three layers in this structure: a perception layer, a control layer, and a planning layer.

For the perception layer, multiple sensors, such as force sensors and position sensors, are utilized to capture the human–robot interaction information. Then, the robot updates the motion output according to the controller in the control layer, respectively. Additionally, to improve the cooperative performance, an obstacle avoidance method and a trajectory adjustment strategy are developed in the planning layer.

### Integrated Framework

**Figure 2:**
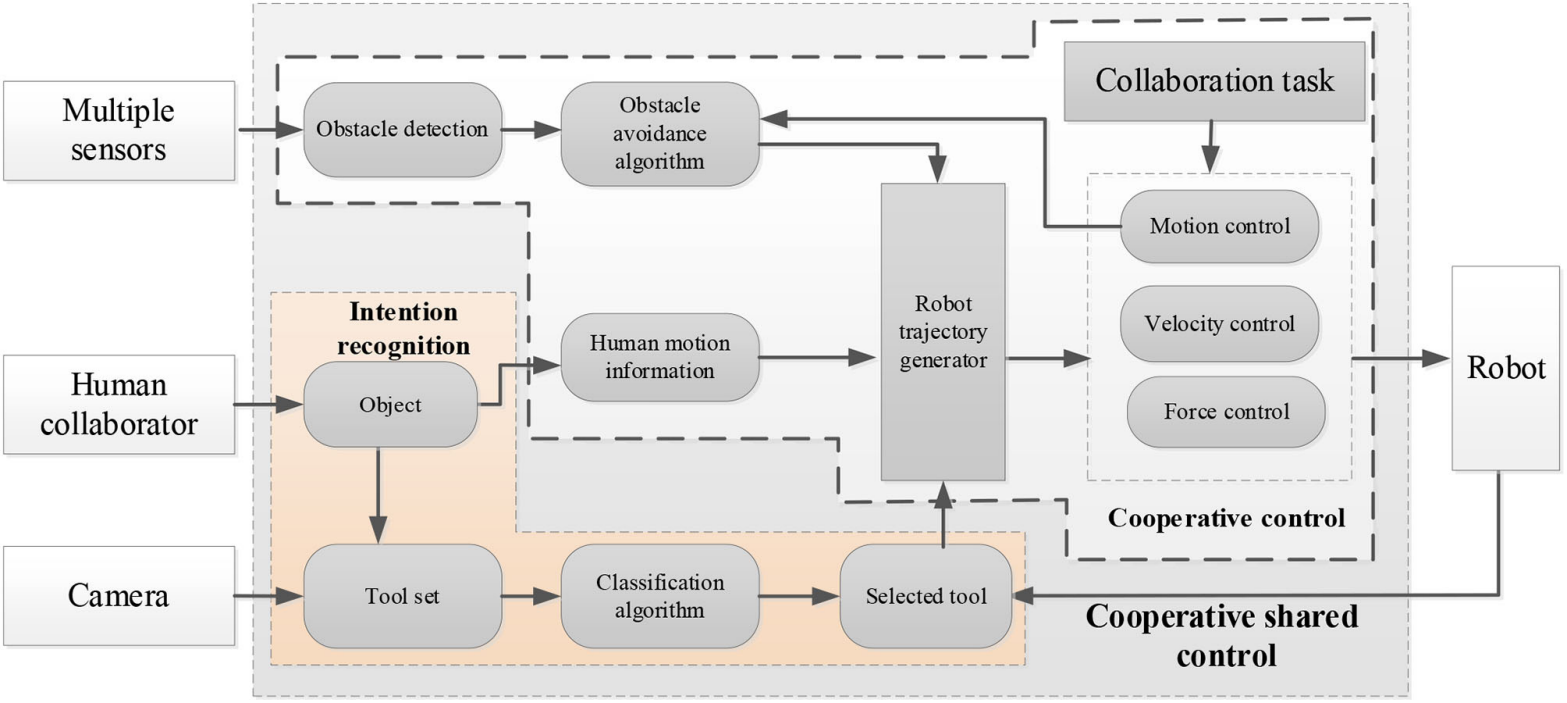
Outline of the proposed scheme.

The outline of the proposed scheme is given in Figure 2. It shows that the proposed approach contains three parts: intention recognition, collaboration and cooperative shared control. They are detailed in the following subsections.

In this article, the computer vision is to identify the operation intention of people through three-dimensional (3D) point cloud and then the robot can identify and judge which kind of screws according to 3D vision, so as to understand the next action of people, such as what tools to take and which kind of screw cap to take. Therefore, the robot can select a suitable tool to cooperate with the human.

The position sensor only generates accumulated errors in a single operation cycle. In this operation cycle, the 3D visual sensor is used to perceive the position of the workpiece in real environment in order to eliminate the accumulated errors of the position sensor. The accumulative error of torque sensor is generated after long time load operation or collision. We eliminate the accumulative error by regular correction. Similarly, we eliminate the accumulated errors of the visual sensors by regular calibration.

### Intention Recognition

We will introduce the recognition of human operation intention through a classification algorithm. The robot can recognize the objects with different accessories and tools in human's hand such as inner hexagon, outer hexagon, and square accessories. By recognizing the objects in the hand, the robot can understand the human intention (i.e., the next planned work of the operator) and then select appropriate tools according to the human intention to help the human carry out the next work procedure. If the operator picks up the outer hexagon screw, the robot can identify it as the outer hexagon screw through 3D point cloud. After the robot identifies the item, according to the process of logic requirements, the operator will read the next step to tighten the outer hexagonal screws and the robot will automatically select the appropriate tool to match the outer hexagonal screws. During the shared time period that the operator picks up the outer hexagon screw and places it in the assembly hole, the robot also prepares the matching tool. The entire process of intention recognition is as follows.

#### Acquisition and Preprocessing of Point Cloud

Coordinates of an object (*x, y, z*) can be obtained through a 3D camera, which includes three matrices: *X*_0_, *Y*_0_, and*Z*_0_. The coordinates represent the positions of the object in X, Y, and Z axes.

We can evaluate the position of the object in the basal coordinates of the robot through a transformation of the coordinates of the camera to control the robot's movement toward the target position.

Considering the impact of illumination, there will be several outliers and noise in the measured point cloud. Generally, this negatively influences the measurement of the object. Therefore, removing such outliers and noise from the point cloud is necessary.

To remove noise and to maintain the edge details of the point cloud, a median filter (MF) is utilized in this section. MF is an optimal filter based on the rule of minimum absolute error. The details of MF are presented in a study by Chen et al. (2018).

#### Binarization

**Figure 3:**
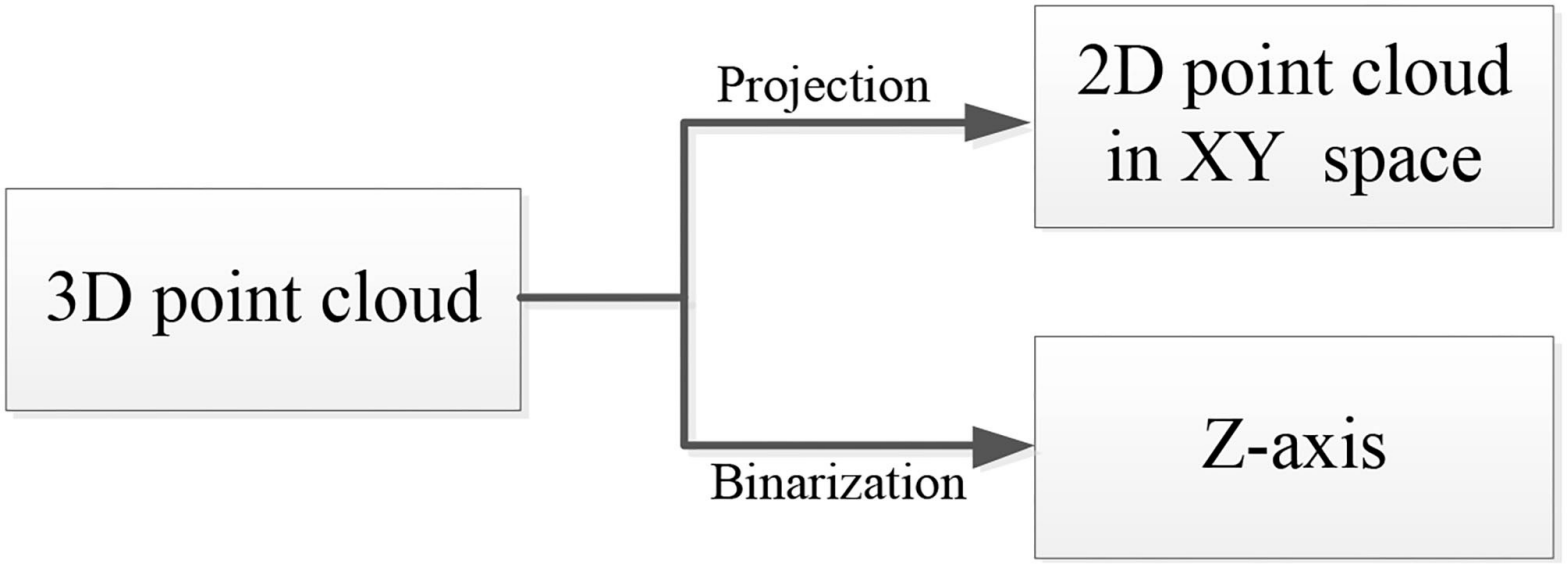
Processing of three-dimensional (3D) point cloud.

To simplify the complexity of point cloud registration, we use binarization in this study. The registration process of the 3D point cloud can be translated to a registration process in a two-dimensional (2D) space through binarization processing (Zou et al., 2018). The detailed process can be observed in Figure 3.

In Figure 3, the 3D point cloud can be translated to a 2D point cloud in the X–Y space and a position along the Z-axis through projection and binarization, respectively.

#### Template Matching

In this study, an error method *D*(*i, j*) is used to estimate the comparability between the template (the trained image sample set) *T*(*m, n*) and an untested template *S*^*i,j*^ (*m, n*) (the test image sample set). It can be presented as:


(1) $$\begin{array}{l} D\left(i,j\right)=\sum_{m=1}^{M}\sum_{n=1}^{M}|S^{i,j}\left(m,n\right)-T\left(m,n\right)|, \end{array}$$

where the minimum of *D*(*i, j*) is the matching position for the template. It should be noted that the larger the *D*(*i, j*), the slower the matching speed and vice versa.

We use the following function to define the comparability of different templates:


(2) $$\begin{array}{l} K_{te}\left(i,j\right)=\left(1-\frac{D\left(i,j\right)}{m\cdot n}\right)\times 100\%, \end{array}$$

where *K*_*te*_(*i, j*) represents the comparability of a template.

Considering the difference between the untested point cloud and the templates, we need to set a threshold to check whether the point cloud is correct. The check equation can be defined as follows:


(3) $$\begin{array}{l} K_{0}=\{\begin{array}{c} 1,\;K_{te}\left(i,j\right)\geq K_{te}\left(i_{0},j_{0}\right)\\ 0,\;K_{te}\left(i,j\right)<K_{te}\left(i_{0},j_{0}\right) \end{array}, \end{array}$$

where *K*_0_ represents the point cloud's correctness. When *K*_*te*_ (*i, j*) ≥ *K*_*te*_ (*i*_0_, *j*_0_), *K*_0_ = 1 implies the point cloud is correct and vice versa.

Based on the abovementioned method, the robot can recognize the objects in the human operator's hand and then can choose a suitable tool to match an object according to the tasks. In this manner, the robot can recognize the human operation intention at the beginning of a mission.

### Cooperative Control

#### Pose Control

**Figure 4:**
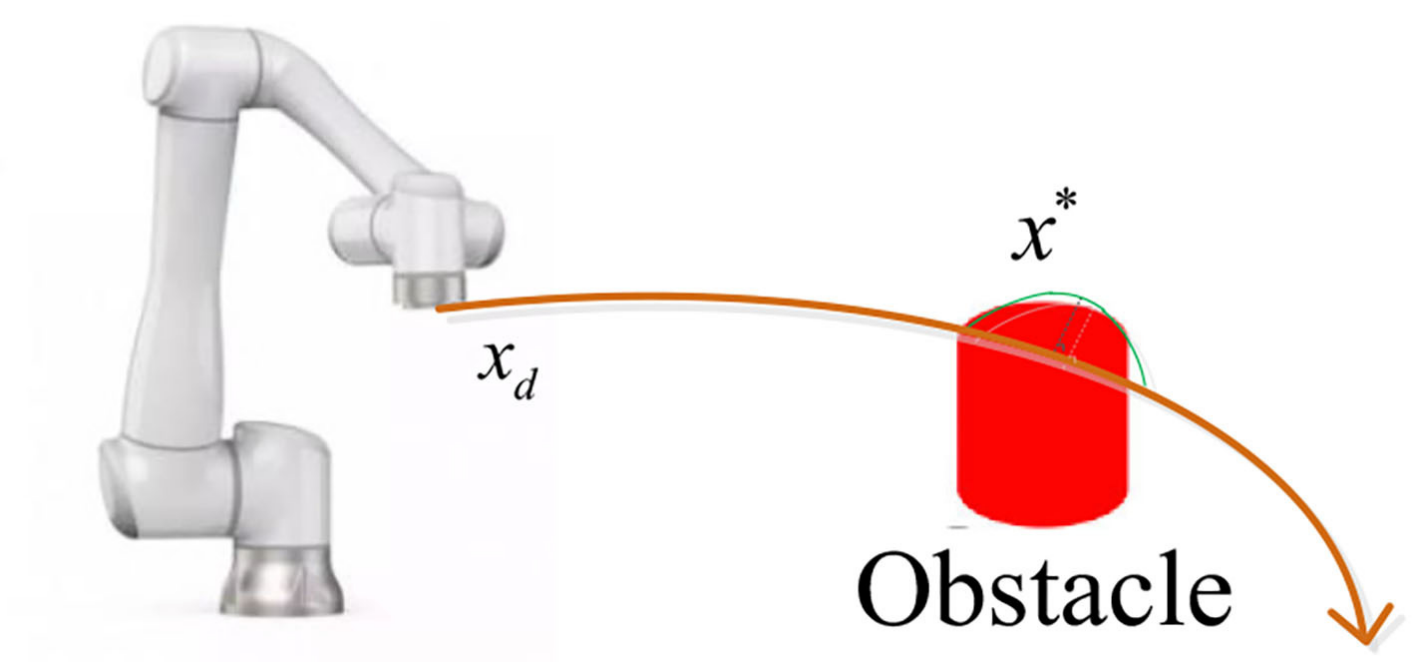
Trajectory generator based on obstacle avoidance for the robot.

Regarding the physical human–robot interaction in a cooperative task, we develop a pose control law to update the robot's trajectory for a safe and effective interaction (Figure 4).


(4) $$\begin{array}{l} x_{d}^{*}=x_{d}+\mu \Delta x, \end{array}$$

where *x*_*d*_ is the predefined trajectory, Δ*x* denotes the adjustment displacement when the robot is affected by the obstacles, and $x_{d}^{*}$ is the desired trajectory after adjustment.

It is noted that the obstacle avoidance method adopts the collision point detection to achieve obstacle avoidance. When the cooperative robot approaches the workpiece, if it collides with the workpiece, the torque of the robot will get a threshold value and the robot stops working at the same time, then the position of the collision point and the direction of force are recorded and defined as the obstacle point. According to the position of the collision point and the direction of force, the robot takes the reverse movement around the collision point. When the robot avoids obstacles, it needs to utilize the information about position of obstacles, the direction of force, the trajectory of movement, the rhythm of workers' operation, and other factors. Redundant safety components are used to ensure human safety such as position sensor, moment sensor, vision sensor, and safety grating.

#### Velocity Control

For a safe interaction, a velocity control method based on a proportional–integral–derivative (PID) controller is proposed. It should be noted that velocity control cannot change the robot's trajectory. Additionally, it is necessary to set a scaling parameter to regulate the robot's speed.

**Figure 5:**
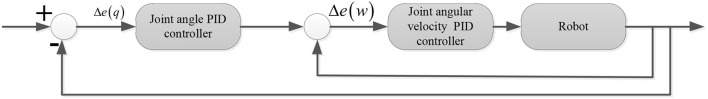
Velocity control of the robot.

As observed in Figure 5, a dual control loop method is proposed to control the joint angle and joint angular velocity.

For the joint angle, an outer control algorithm based on a PID controller is developed as:


(5) $$\begin{array}{l} q_{i}=K_{qp}\left(q_{d}-q_{i}\right)+K_{qi}\int dq_{i}+K_{qd}\frac{dq_{i}}{dt}, \end{array}$$

where *K*_*qp*_, *K*_*qi*_, and *K*_*qd*_ denote the control parameters of the joint angle and *q*_*d*_ and *q*_*i*_ are the desired joint angle and the actual joint angle, respectively.

For the joint angular velocity, the controller is designed as:


(6) $$\begin{array}{l} w_{i}=K_{wp}\left(w_{d}-w_{i}\right)+K_{wi}\int dw_{i}+K_{wd}\frac{dw_{i}}{dt}, \end{array}$$

where *K*_*wp*_, *K*_*wi*_, and *K*_*wd*_ denote the control parameters of the joint angular velocity and *w*_*d*_ and *w*_*i*_ are the desired joint angular velocity and the actual joint angular velocity, respectively.

Considering the impact of the robot's speed, such as insecurity, we propose a scaling parameter to regulate the joint angle and joint angular velocity as follows:


(7) $$\begin{array}{l} q_{control}=\left(1-\varepsilon \right)q_{i}, \end{array}$$

(8) $$\begin{array}{l} w_{control}=\left(1-\varepsilon \right)w_{i}, \end{array}$$

where *q*_*control*_ and *w*_*control*_ are the control joint angle and control joint angular velocity after regulation, respectively, and ε ∈ [0, 1] is a scaling factor.

#### Force Control

The dynamic model of the robot can be given as:


(9) $$\begin{array}{l} MẌ+BẊ=u+F_{set}+F_{d}, \end{array}$$

where *M* is the positive definite inertia matrix and *B* denotes the positive definite damping matrix. *F*_*d*_ is the disturbance force, *u* represents the robot's control variable, and *F*_*set*_ is the setting force of the robot applied to the tools. In Equation (9), u is a control force of the robot; it is utilized in task/motion space and can be transformed into the torque in the joint space based on the robot kinematics.

For torque control, the value of u is limited within [0, *F*_*set*_].

In this study, the robot controller is designed as:


(10) $$\begin{array}{l} u=M{Ẍ}_{d}+B{Ẋ}_{d}. \end{array}$$

It is clear that the stability of force control can be guaranteed.

During the cooperative control period, the robot realizes space and time sharing with the operator by adjusting the control parameters such as position, speed, and torque. The actions of the operator include: holding spare parts (screws), placing screws, selecting remaining parts (gaskets, screw caps), installing remaining parts, selecting tools, tightening operations, putting back tools, and putting back truck parts. The actions of the robot include: moving to the starting point, taking photos, identifying intentions, selecting tools, calculating the assembly point, running to the assembly point, fixing screws, maintaining torque, detecting rotation direction and torque, returning to the shooting point, and returning to the starting point.

In the cooperation between the robot and the operator, the robot is responsible for controlling the fixing and rotating torque of one end of the screw with its precise position control and torque control. The operator is responsible for loosening or tightening the other end of the screw. The operator's release feedback to the end-degree of freedom of the robot is the counterclockwise torque of rotation. The operator's tightening operation feedback to the end-degree of freedom of the robot is the clockwise rotation moment. The robot can identify whether the operator is tightening or loosening the operation by the direction of the end torque. In practical applications, when there are multiple screws of the same size to be assembled, the robot works one by one according to the origin of the image collected by the 3D camera. If there are some screws that do not need to be operated, the operator can block some screws with his hand or other objects during the positioning stage of the robot's image collection and the robot will operate one by one according to the screws revealed in the image collection. Therefore, the safety components for robot and operator cooperation are redundant.

## Results

To verify the performance of the proposed method, a cooperative assembly task for flexible manufacturing is performed. In this experiment, we will examine the intention recognition algorithm, obstacle avoidance, and cooperative control.

### Experimental Setup

In this article, a composite robot is used; it is a new robot category composed of mobile robot and cooperative manipulator, combined with our 3D camera, which installed at the end of a 6-DOF cooperative robot. Therefore, it can perform variety of functions such as the human hand (robot arm), foot (mobile robot), and eye (3D camera). In HRC, its advantages include: (a) to choose a more suitable stopping point for HRC; (b) breaking through the limitation of the arm span size of the mechanical arm, expanding the scope of operation; (c) planning the operation trajectory in HRC; (d) identify the parts to be assembled accurately; (e) understanding of human intentions; (f) collision perception and control during human–robot operation; and (g) meet the safety standards of human–robot cooperation.

**Figure 6:**
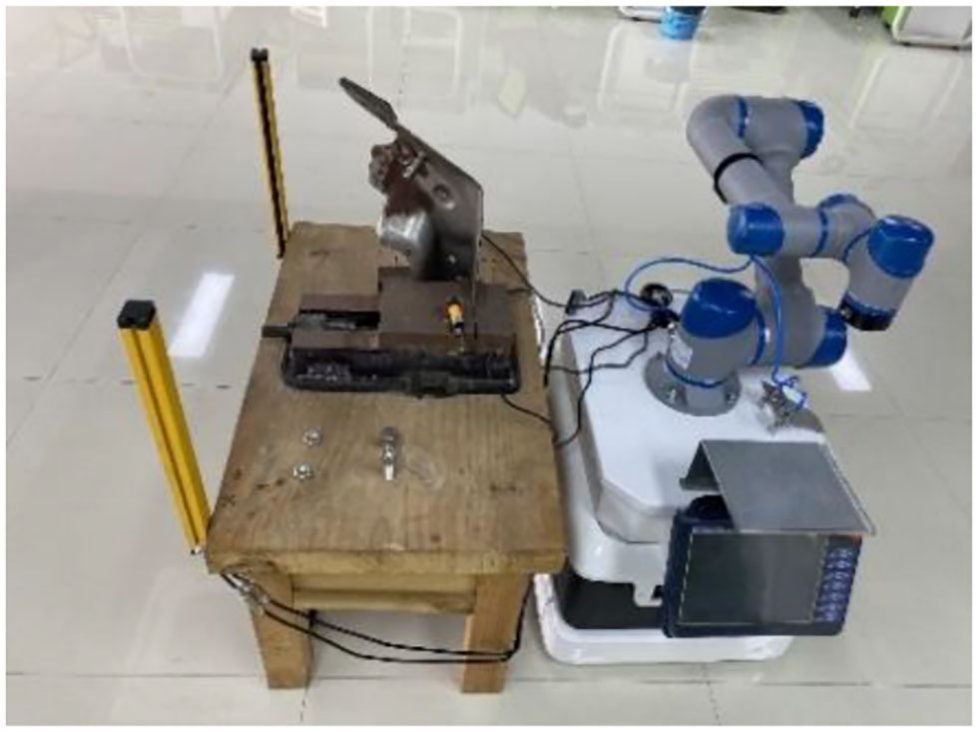
Cooperative assembly task in the experiment.

As shown in Figure 6, a human operator and a collaborative robot are used to perform the flexible assembly task of truck fitting.

**Figure 7:**
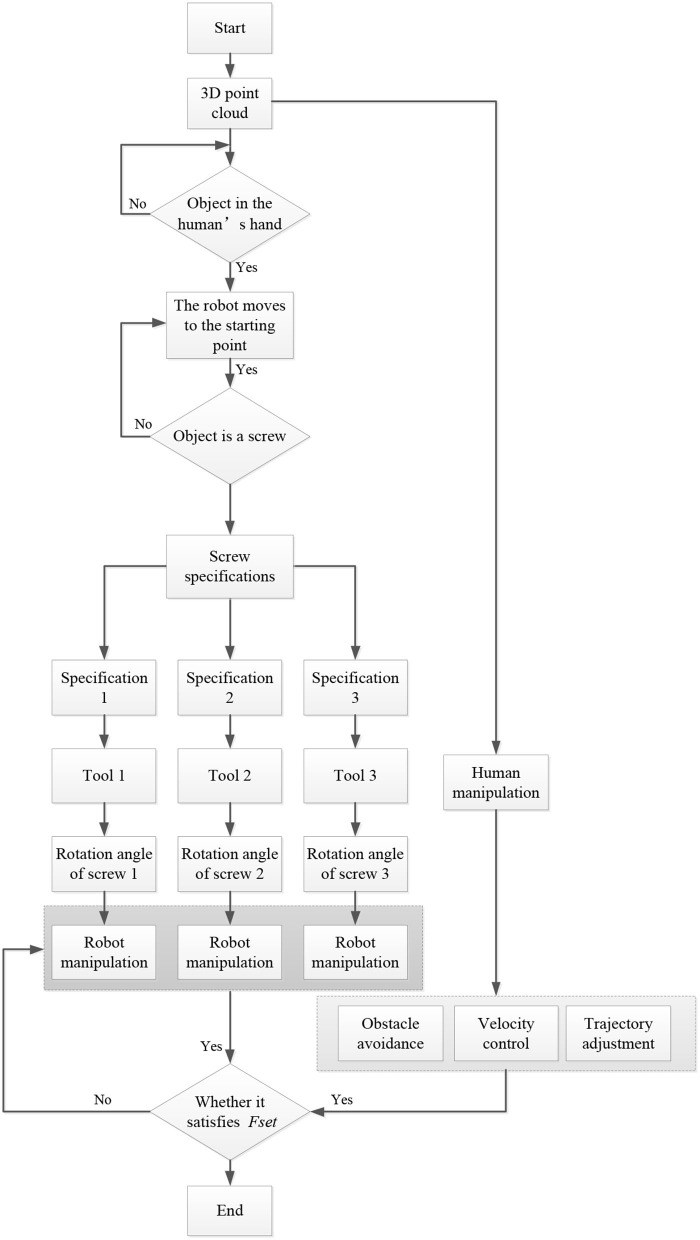
Flowchart of the experiment.

**Figure 8:**
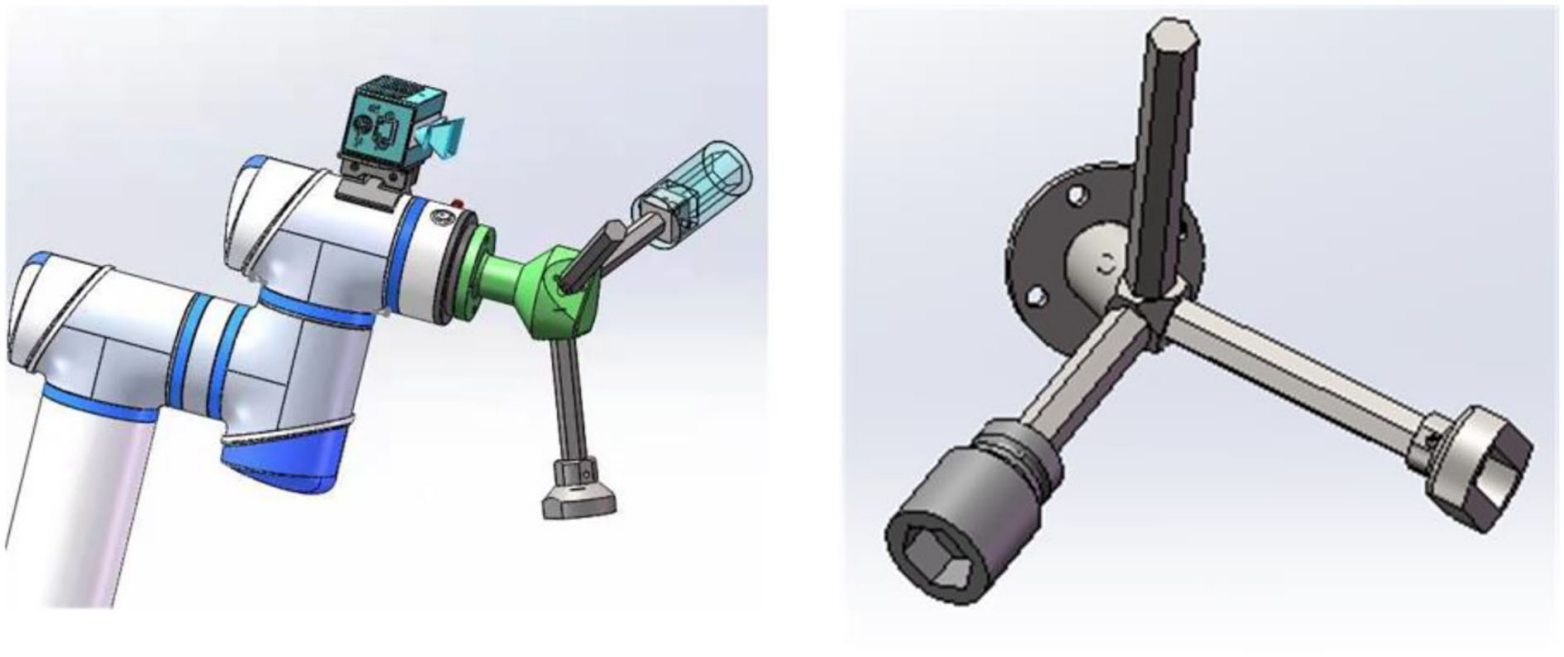
Multipurpose tool installed at the robot's end effector.

The flowchart of the cooperation process is given in Figure 7. In this experiment, the robot uses a 3D camera to create a 3D point cloud model of the workpiece to be assembled in the application scene. First, the operator picks up an assembly part such as a square screw. At the same time, the robot moves to the starting point and begins to classify and identify the size of the assembly part in the hand. The robot identifies the specific specifications of the parts through the classification algorithm and then combines with the requirements of the assembly process to identify the operation intention of the operator (i.e., the operator plans to carry out the next work). The robot automatically selects the appropriate tools matching the parts and screws together with the operator. It should be noted that there are three different tools to match the screws (Figure 8).

The shared time between the operator picking up the part (screw) and placing the screw in the target position of the truck part. The robot performs three tasks successively such as identifying spare parts, understanding the operator's intention, and selecting matching tools. From the moment, the operator places the screw at the target position of the truck fitting, the robot sets the screw with the appropriate tool, and compacts it and maintains the set torque of rotation. The robot senses the rotating force in the opposite direction by the terminal degree of freedom and determines whether the worker is tightening or loosening the work by the direction of the rotating force in the opposite direction. Determine whether the screws are tightened by the amount of the counterrotating force. At the same time, the operator selects the remaining parts of the task (such as gasket, screw cap) to tighten the other end of the screw. In the shared time and space of cooperative tightening operation, the cooperative robot exerts two forces on the assembly workpiece: one robot is the pressing force of fixed action and another robot is the rotating force of tightening action. In the process of screw tightening, the rotating force generated by the worker is transmitted to the cooperative robot through the screw column and the cooperative robot can judge whether the screw is tightened by perceiving the rotating force in the opposite direction.

It should be noted that using obstacle avoidance algorithm and cooperative control method, the robot needs to adjust the trajectory to cooperate with the operator. In actual assembly operations, in order to select a comfortable operation behavior (such as arms close, arms crossed, and single-arm full bend), the operator's two arms may be on the trajectory of the robot. At this time, the robot will choose to avoid obstacles and circumnavigate according to the direction and magnitude of the impact torque on the robot end effector. The robot then chooses the trajectory in the opposite direction to circumnavigate to the target point.

### Results Analysis

#### Human Operation Intention Recognition

**Figure 9:**
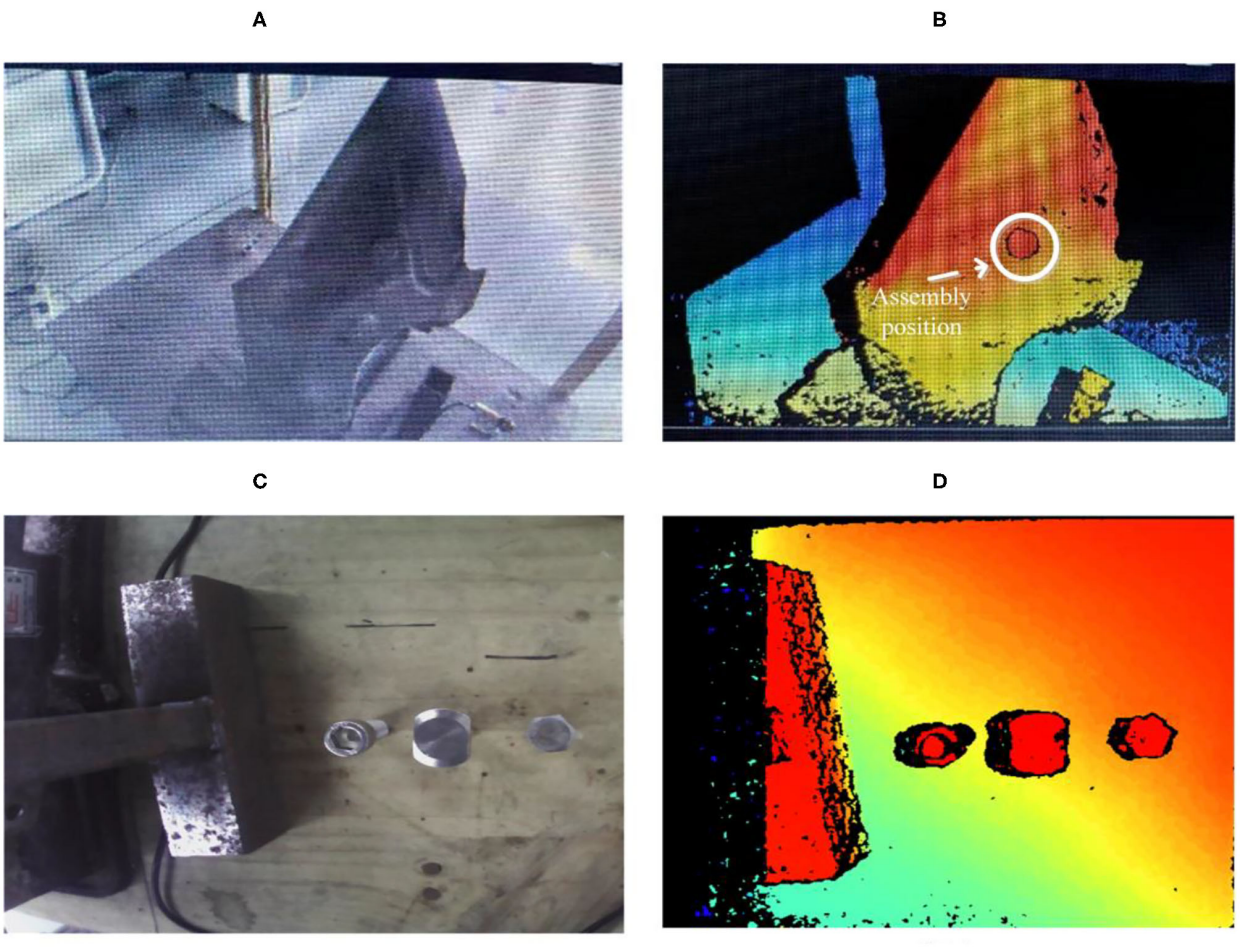
Modeling of the assembly environment. **(A,B)** Are the actual picture and 3D point cloud picture of truck parts respectively. **(C,D)** Are the actual picture and 3D point cloud picture of screws respectively.

Figure 9 shows the assembly task's work environment modeling. Figures 9A,C show the actual truck fitting and the screws, respectively. Based on the 3D point cloud technology, we can model the virtual environment of truck fitting and screws, as shown in Figures 9B,D, respectively. The white circle is the assembly position for the truck fitting task. Through the 3D camera, the robot collects the point cloud of the assembly parts (three types of screws) and matches it with the standard three types of point cloud template (outer hexagon, inner hexagon, and square). It senses what parts the operator is holding and the specifications of the parts and then understands the operator's next work plan based on the assembly process.

**Figure 10:**
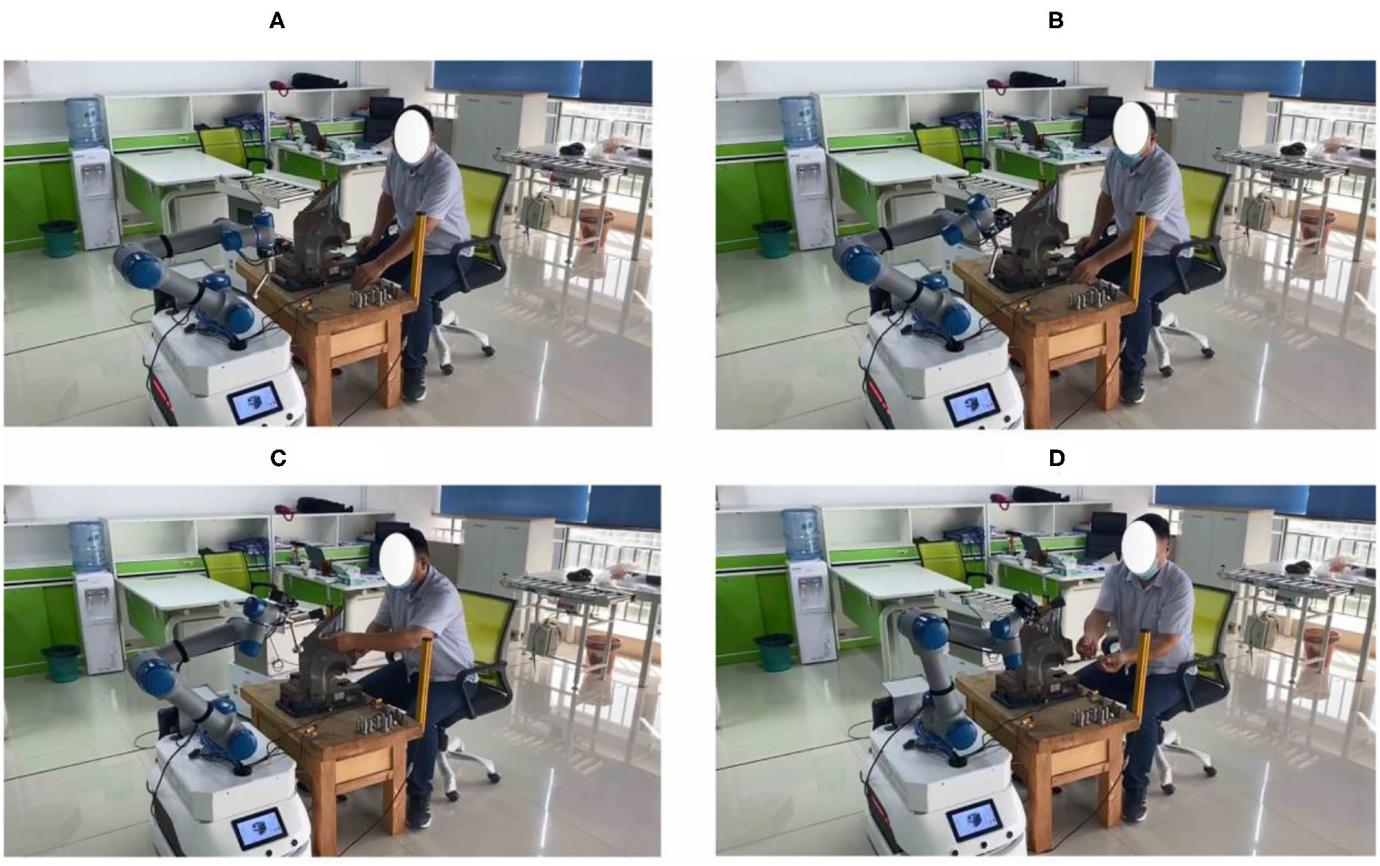
Human operation intention recognition. **(A)** Indicates that the robot reads the instruction and starts. **(B)** Means to understand the intention of the operator. **(C)** Indicates the selection of qualified workpiece. **(D)** Represents collaborative assembly.

In Figure 10, the robot can recognize the human operation intention based on a classification algorithm. We can observe that the robot classifies the screw in the human operator's hand when the assembly task is executed and then selects a suitable tool to help the human operator.

#### Obstacle Avoidance

**Figure 11:**
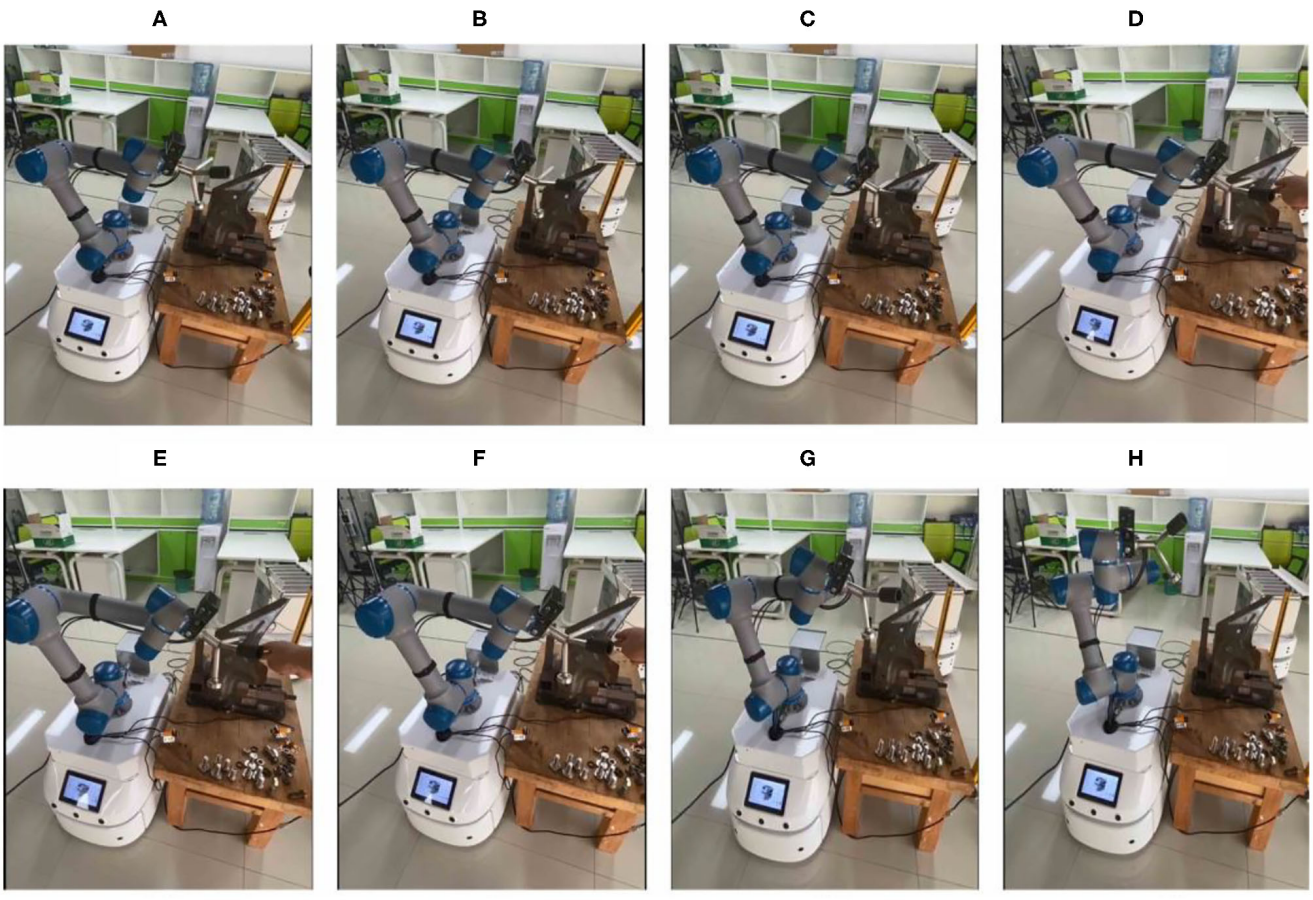
Robot's obstacle avoidance process. **(A–C)** Present Normal operation track. **(D–H)** Present Collision track.

Figure 11 presents the entire obstacle avoidance process. Figures 11A–C present the robot movements for truck fitting. When the robot is close to the truck fitting, it will regulate its speed and trajectory to avoid the truck fitting location. In Figures 11D–H, when the robot encounters the human, it will stop moving and then update its trajectory based on a predefined trajectory. It should be noted that the maximum speeds of the robot are set to 0.45, 0.3, and 0.15 m/s, respectively. The robot can regulate its speed according to the different conditions.

#### Cooperative Assembly

##### Simulation

**Figure 12:**
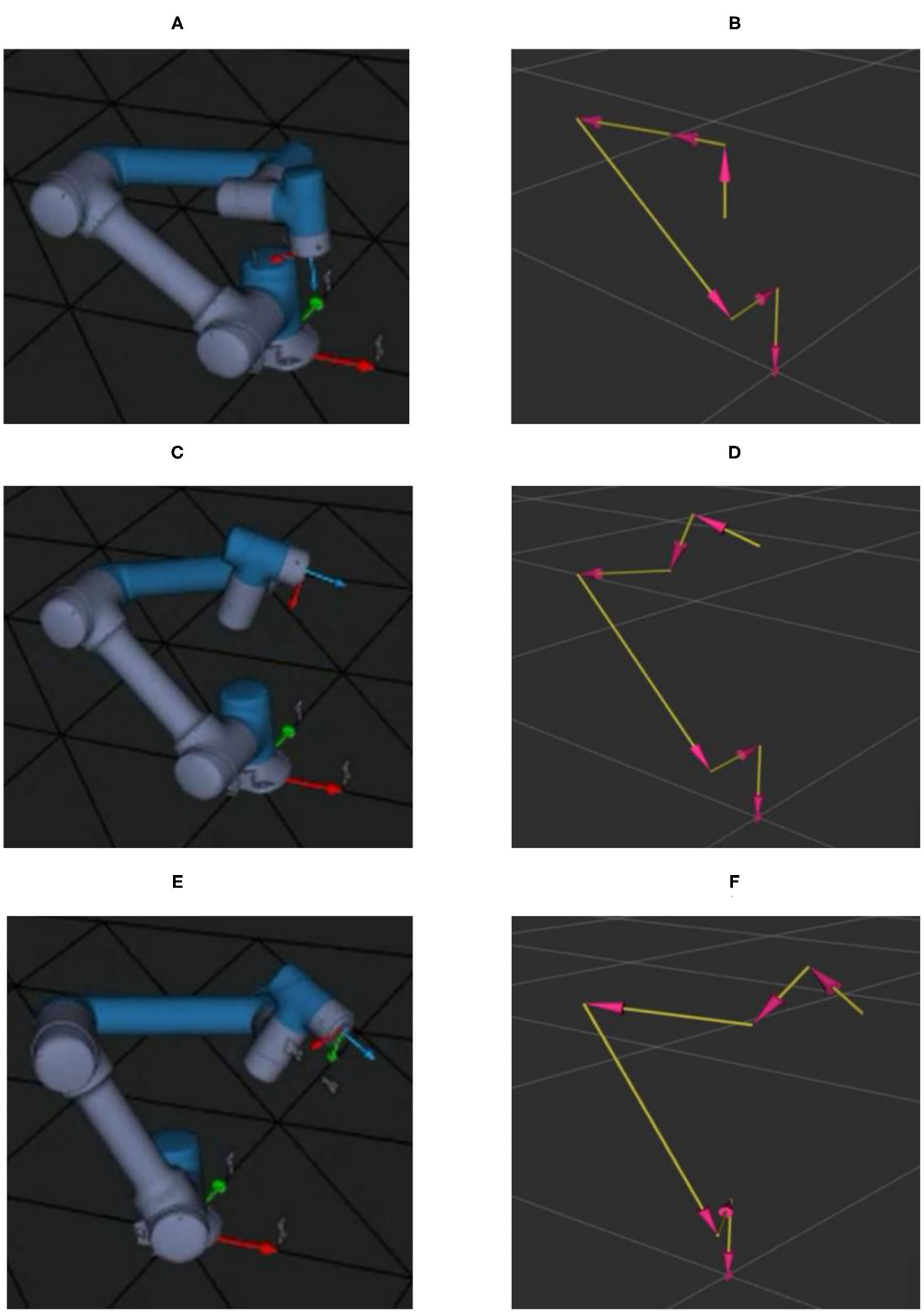
Simulation process of cooperative assembly. **(A,B)** Represent the initial position. **(C,D)** Represent robot photo pose. **(E,F)** Represent collaborative assembly pose.

In order to guarantee the performance of cooperative assembly, a simulation is performed. As shown in Figures 12A,C,E are 3D model of the cooperative robot and (Figures 12B,D,F) are parameters model of the cooperative robot.

Figures 12A,B are the initial state of the robot. In this stage, before the beginning of the collaborative assembly, the process includes the preparation of 3D camera and the completion of initial information monitoring of the collaborative assembly.

In Figures 12C,D, after receiving the operator's hands to assembly screws information for the robot, the robot runs from the starting position to take photos position and then through the 3D camera's perception on the screw assembly area; if the operator has place the screws on the artifacts, 3D camera further recognizes the specifications of the screw and identifies the specifications of the screw. Then, the robot selects the suitable tool to match the screw according to the operator's intention.

Figures 12E,F represent the pose of human intention recognition with perception and the pose of collaborative assembly, respectively.

**Figure 13:**
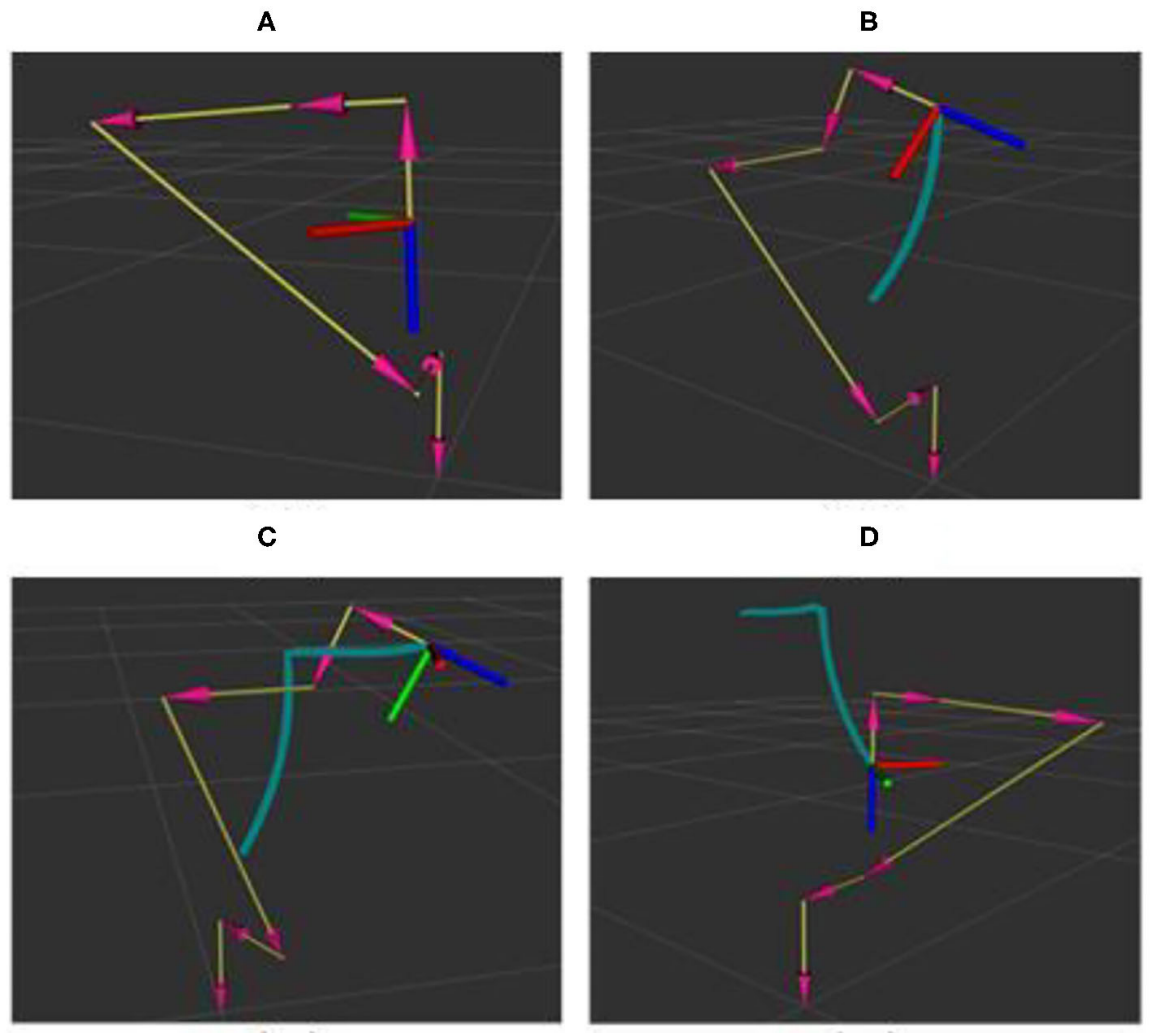
Desired trajectory of the robot in the assembly task. **(A–D)** Presents the trajectories of robot starting, photographing, assembling and returning to the starting point respectively.

Figure 13 shows desired trajectory of the robot in the assembly task. Figures 13A,D represent the initial position and ending position, respectively. It is noted that there is the same place for the initial position and the ending position. Figure 13B denotes the position to take a picture for the 3D camera. Figure 13C is the assembly pose for the robot.

##### Experiment

**Figure 14:**
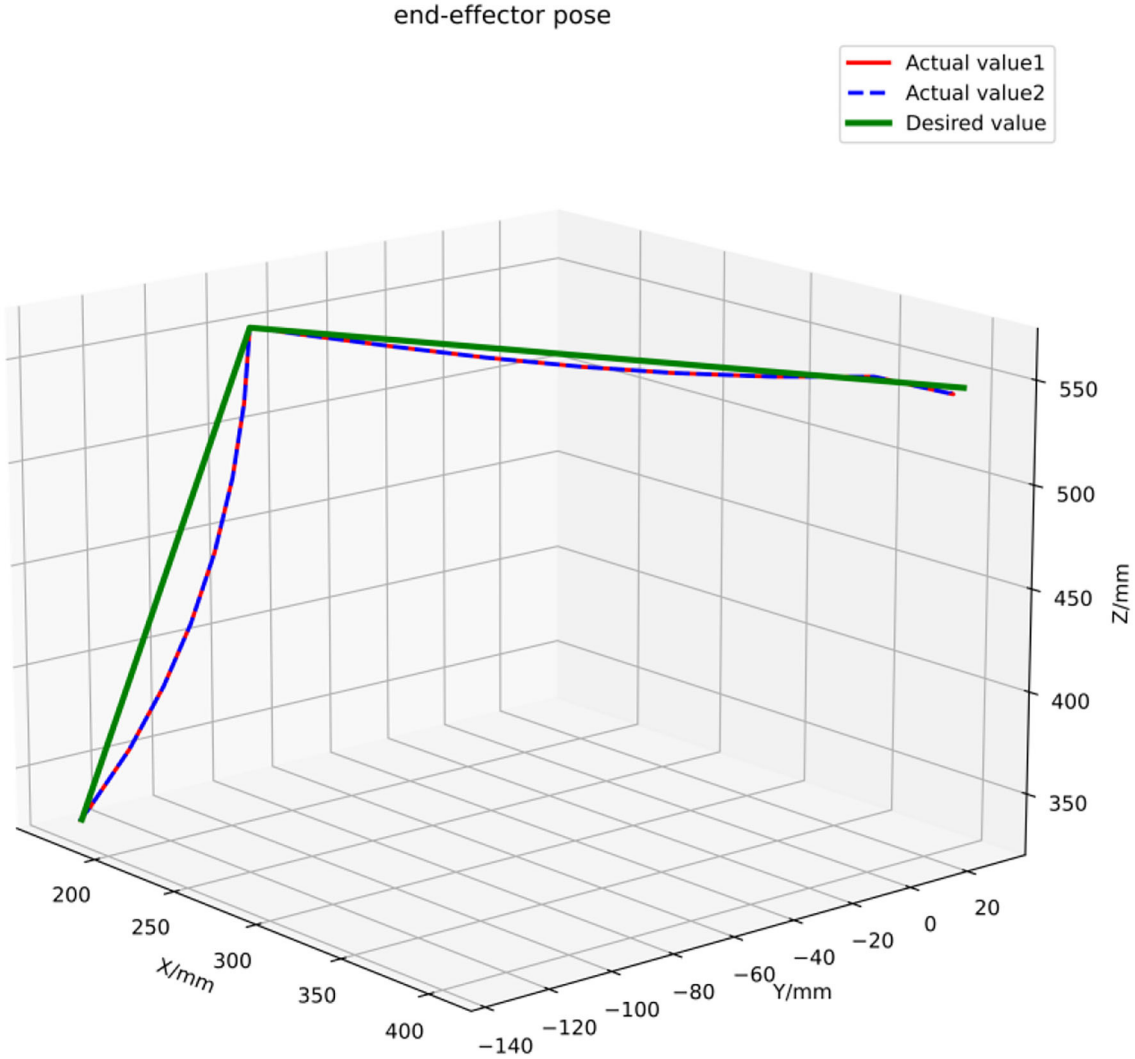
Actual trajectory of the robot in the assembly task in 3D space.

**Figure 15:**
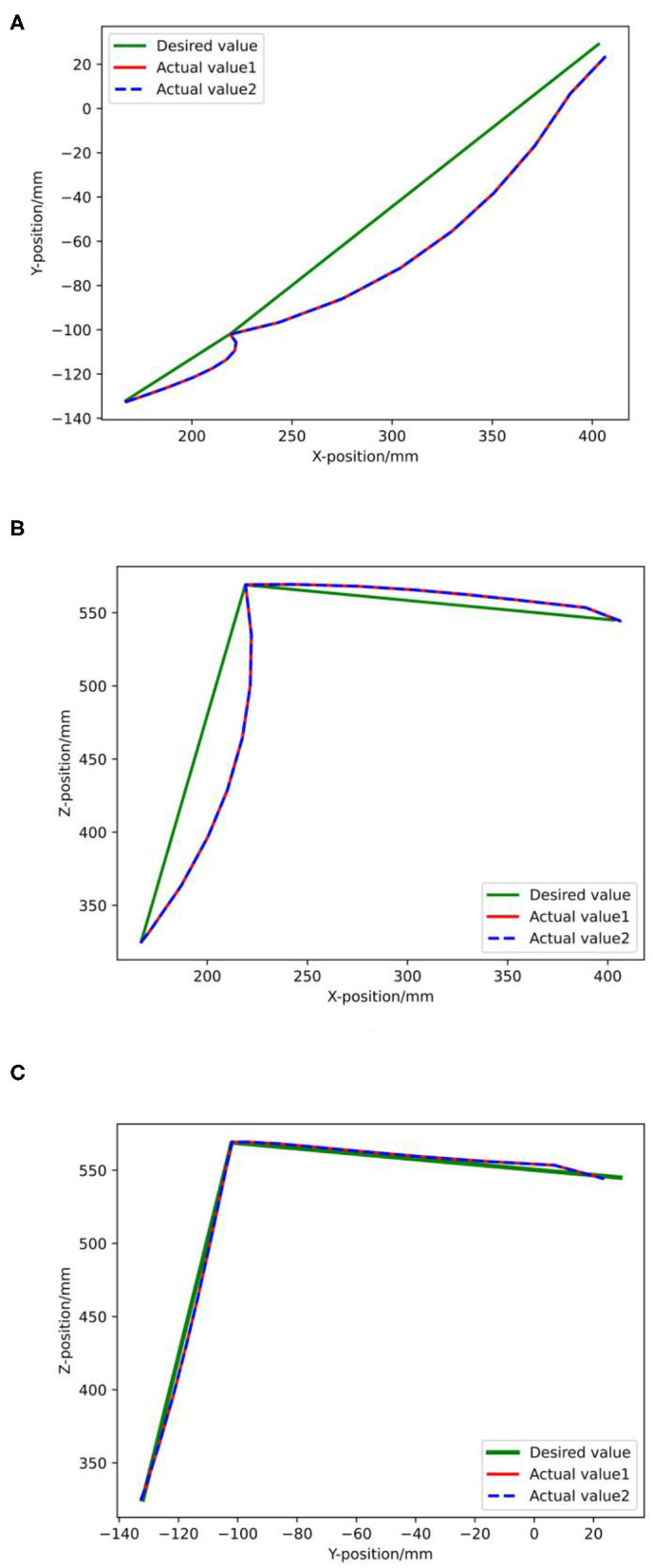
Actual trajectory of the robot in the assembly task in X-Y-Z axes.

Figure 14 denotes the actual trajectory of the robot in the assembly task in the 3D space. Figure 15 shows actual trajectory of the robot in the assembly task in X, Y, and Z axes. It can be seen that green curve is the desired trajectory and the red and the blue are the actual trajectories for two assembly experiments.

The expected trajectory of the robot end effector is a trajectory connected by starting point, camera point, assembly point, and end point, assuming that there is enough space for the robot to run above the workpiece. The robot runs from the starting point to the shooting point, then to the assembly point, and then back to the shooting point and the ending point. The starting point and the ending point are the same position, which refer to the position where the mobile robot is parked beside the station. According to the size of the workpiece, different spatial positions and poses are set for the photo point. The setting is based on the normal direction perpendicular to the workpiece working surface, 350 mm away from the workpiece working surface. According to the data collected by the 3D camera, the robot determines the coordinates of the workpiece relative to the starting point of the robot and the position of the robot end effector corresponding to the modified coordinates is the coordinate of the assembly point in the trajectory.

**Figure 16:**
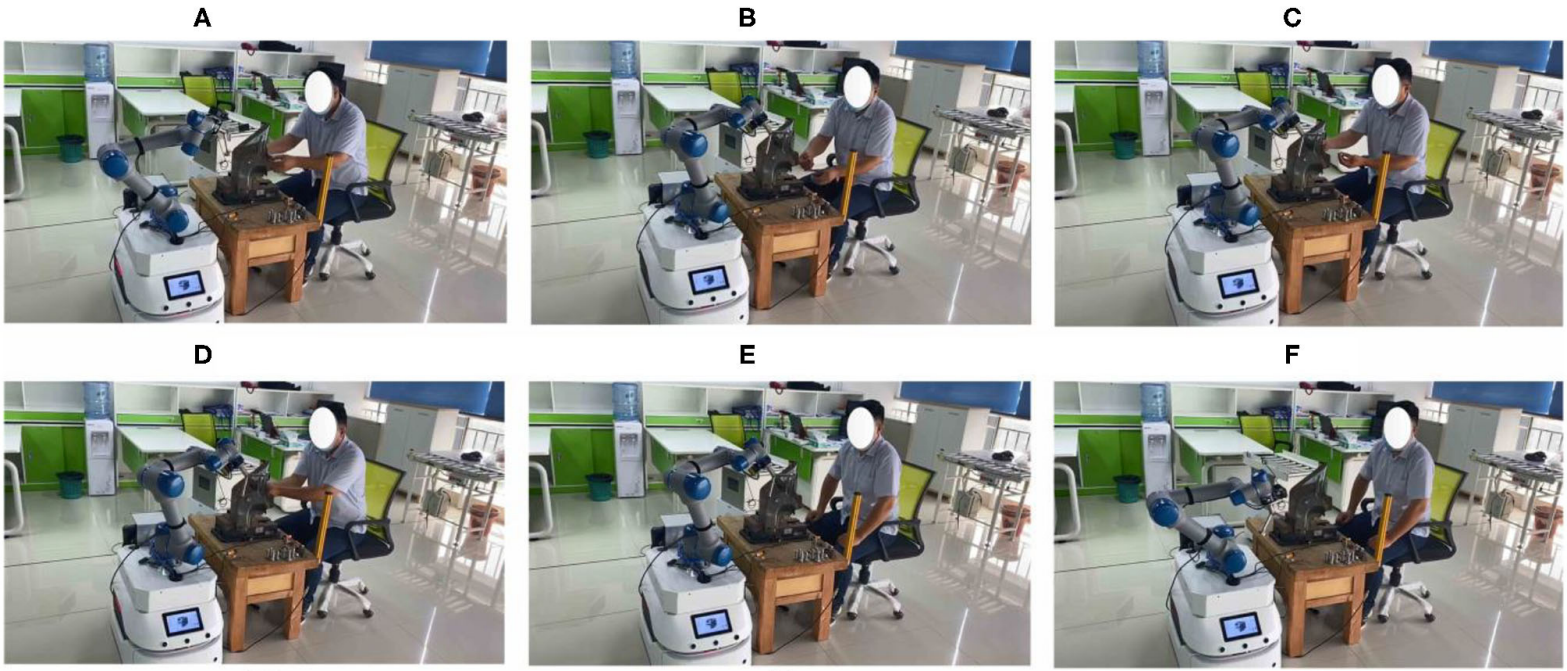
Human-robot cooperative assembly. **(A)** Indicates Selection of adaptation tools. **(B)** Indicates pressure. **(C)** Indicates the assembly. **(D)** Indicates Induction torque. **(E)** Indicates end. **(F)** Indicates back to the starting point.

**Figure 17:**
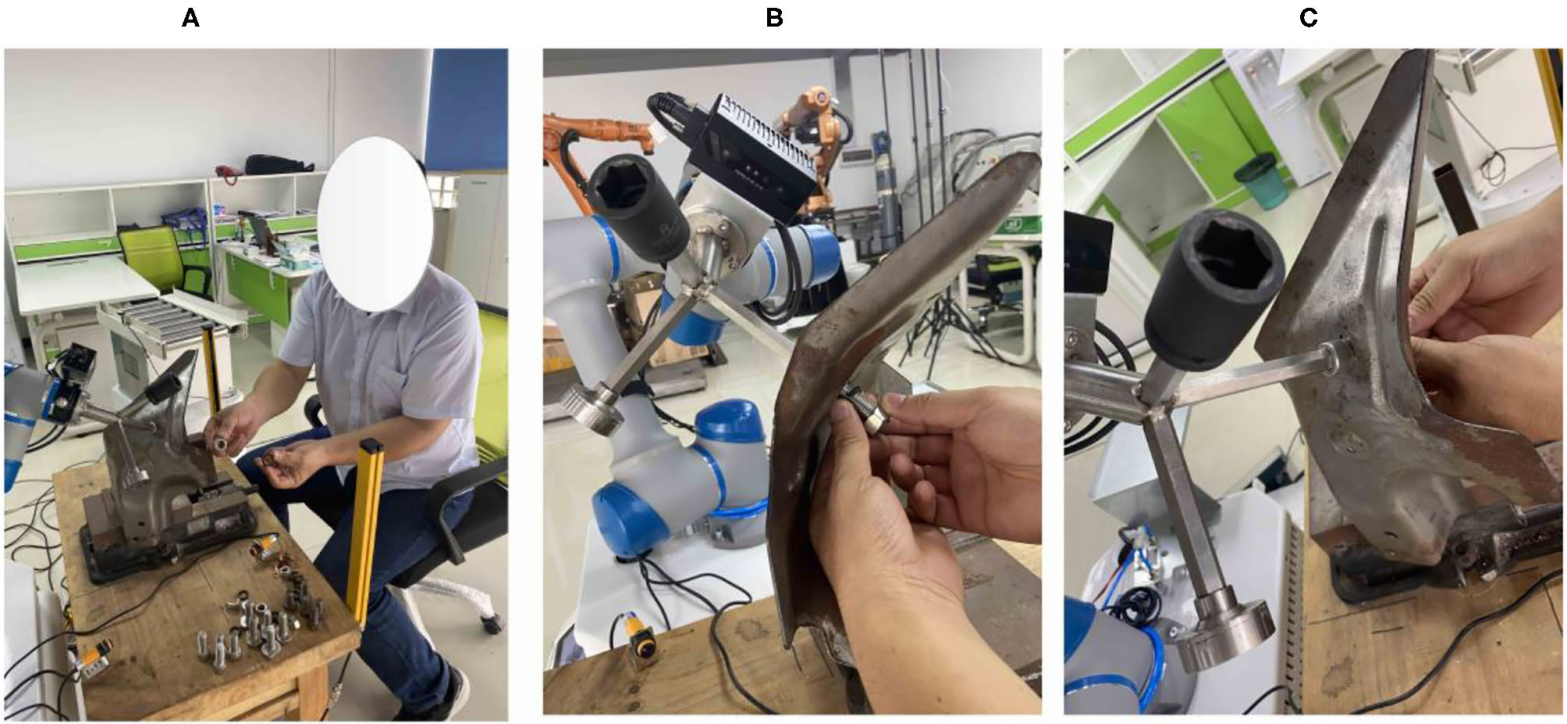
Detailed process of human-robot cooperative assembly. **(A)** Indicates that adaptation tools are selected. **(B)** Represents assembly. **(C)** Stands for induced torsion.

Figures 16, 17 describe the cooperative process between the robot and the human operator for the assembly task. Based on the human operation intention recognition and obstacle avoidance, the robot selects a suitable tool to help the human finish the assembly task. It can be observed that the robot holds one end of the screw when the human works in Figures 13A–D, 14. When the human finishes the task and the applied force is equal to *F*_*set*_, the robot will move to the starting point.

According to the track line in Figure 15, the actual track at the starting point and the photo point overlapped with the expected track point and there was a distance between the actual track and the expected track at the assembly point because in the actual tightening screw assembly, the screw would have some rotation and translation movements.

**Figure 18:**
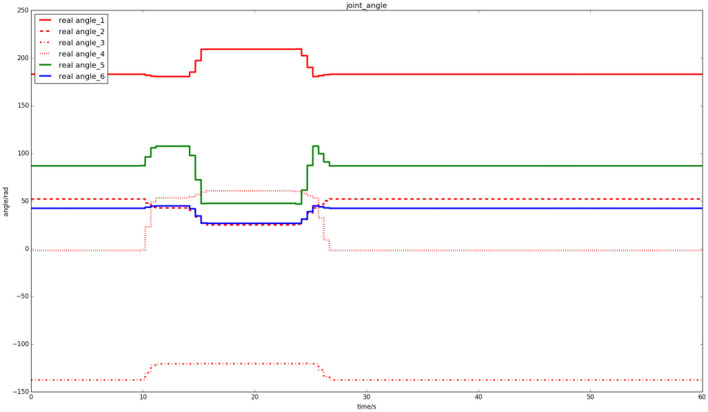
Joint trajectories of the robot in cooperative assembly task.

Figure 18 shows the joint trajectories of the robot in cooperative assembly task. It can be seen that the robot performs the task with the human in the first 10–30 s.

##### Statistical Analysis

In order to further verify the performance of proposed cooperative shared control method, we perform the experiments under two different experimental situations: cooperative assembly with or without cooperative shared control.

In Table 1, we have performed 10 times cooperative assembly under the condition of without cooperative shared control. Although robots and workers work in the shared space, they are relatively independent and work according to their own beats. The main functional modules are trajectory planning, speed planning, collision detection, visual positioning, safety detection, and so on. The identification of workpiece, the understanding of human intention, and the collaborative assembly in space and time have not been solved. It can be seen that only two times are successful to complete the task. The assembly period requires a fixed time of at least 17.5 s for successful manipulation, which is the limit of operation. If the time of operators is compressed, the assembly will fail due to insufficient time. Additionally, if the running time of the cooperative robot is compressed, it will collide with people and the task will be failed. In this sense, it is essential to utilize the cooperative control strategy such as cooperative shared control for the human–robot cooperation tasks.

**Table 1:** Cooperative assembly without cooperative shared control.

**Time for the camera (s)**	**Time for the human operator (s)**	**Time for the robot (s)**	**Task is successful or not?**	**Completion time (s)**
3	7.8	6.5	Yes	17.3
3	6.8	6.5	No	16.3
3	7.4	6.5	No	16.9
3	7.2	6.5	No	16.7
3	7.5	6.5	No	17
3	7	6.5	No	16.5
3	7.3	6.5	No	16.8
3	7.5	6.5	No	17
3	8.2	6.5	Yes	17.7
3	7.1	6.5	No	16.6

Table 2 shows the performance of cooperative assembly with cooperative shared control. It can be seen that there are no failure for the cooperative assembly task by using the cooperative shared control method. The assembly time was not fixed and the average time after multiple tests was 18.9 s. In comparison, although there is a little longer than without cooperative shared control, the success rate is reaching 100%. In addition, the running speed of the robot can be improved; the operation time of the cooperative robot also can be compressed, so the entire assembly time can be reduced. By using the cooperative shared control method, increasing the speed of the cooperative robot will not lead to collision.

**Table 2:** Cooperative assembly with cooperative shared control.

**Time for the camera (s)**	**Time for the human operator (s)**	**Time for the robot (s)**	**Task is successful or not?**	**Completion time (s)**
2	10.5	6.5	Yes	19
1.5	11	6.5	Yes	19
2.5	12	6.5	Yes	21
2.6	10.3	6.5	Yes	19.4
2.4	9.8	6.5	Yes	18.7
2.2	10.2	6.5	Yes	18.9
2.7	9.7	6.5	Yes	18.9
1.8	9.5	6.5	Yes	17.8
1.8	10.2	6.5	Yes	18.5
2.1	10	6.5	Yes	18.6

## Discussion

Traditionally, the robot works with the humans in a same workspace with noninterference with each other. Based on sharing a workspace, the sharing of workspace and time needs to consider the task allocation and collaboration according to the assembly technology. In order to improve the efficiency of task execution and natural interaction for the HRC tasks, shared control is proposed. In this study, a cooperative shared control scheme based on intention recognition is developed for flexible assembly manufacturing. For a smooth interaction and synchronous operation, we propose a robot motion control method to deal with obstacle avoidance and cooperative operation. Additionally, a human intention algorithm is proposed for the robot to match the human's operation through the sharing of workspace and time. To verify the developed approach, a simple assembly task of truck fitting is performed. Indeed, shared control approach is suitable to utilize in multiple HRC working scenarios. In the design of shared control, the versatility and scalability should be taken into consideration, especially in unstructured interaction environment. Based on actual application scenarios, this solution can be applied to other scenarios such as product quality detection and coronavirus disease 2019 (COVID-19) sample collection. For example, in product quality testing, collaborative robots and cameras are necessary components and sensors related to qualified indicators need to be added. The sensor data of qualified index is an important reference for the robot to judge the product quality, which affects the content and process of the robot's subsequent operation. In the future, we will test human operation intentions in multiple working scenarios in order to evaluate the effectiveness and generality of shared control such as the applicability of the scheme in product size measurement, reliability testing, finished product packaging, and other practical scenarios. Additionally, we will actively consider the humanoid control and put the human stiffness transfer into the robot to enhance the performance.

## Conclusion

This article proposed a scheme based on cooperative control and intention recognition and provides a feasible solution for flexible assembly manufacturing. It should be noted that there are multiple algorithms to recognize human operation intention such as from human EMG signals. However, this method makes it difficult to accurately estimate the human control intention. Our approach is based on robot vision and can be extended to other operational areas. Furthermore, we proposed a cooperative shared control algorithm to solve the issue of workspace and time sharing between a robot and a human. Although we only tested a simple assembly task, the proposed scheme provides an example for flexible manufacturing. One weakness of our developed approach is in terms of estimating the human arm's stiffness in the cooperation process owing to the difficulty in accurately calculating it. Furthermore, the shared control method may limit the flexibility of cooperative control for more complicated assembly tasks. In the future, we will consider stiffness control for HRC.

## Data Availability Statement

The original contributions presented in the study are included in the article/supplementary material, further inquiries can be directed to the corresponding author.

## Author Contributions

GZ and JL: human-robot collaboration system, experiments, manuscript writing, original draft, writing, review and editing, and funding acquisition. SX, SZ, and GZ: methodology. SX and GZ: results analysis. All authors read and edited the manuscript, and agrees with its content.

## Funding

This article was funded by the Hunan Natural Science Foundation Program (2021JJ40610), the Key Area Research and Development Program of Guangdong Province (2020B0101130012), and the Foshan Science and Technology Innovation Team Project (FS0AA-KJ919-4402-0060).

## Conflict of Interest

The authors declare that the research was conducted in the absence of any commercial or financial relationships that could be construed as a potential conflict of interest.

## Publisher's Note

All claims expressed in this article are solely those of the authors and do not necessarily represent those of their affiliated organizations, or those of the publisher, the editors and the reviewers. Any product that may be evaluated in this article, or claim that may be made by its manufacturer, is not guaranteed or endorsed by the publisher.
